# Use of Meloxicam or Ketoprofen for Piglet Pain Control Following Surgical Castration

**DOI:** 10.3389/fvets.2018.00299

**Published:** 2018-11-26

**Authors:** Abbie V. Viscardi, Patricia V. Turner

**Affiliations:** Department of Pathobiology, University of Guelph, Guelph, ON, Canada

**Keywords:** analgesia, animal welfare, castration, piglet, pain assessment, NSAID

## Abstract

Surgical castration of piglets is performed routinely on commercial pig farms, to prevent boar taint and minimize aggression. While this procedure is known to be painful, piglets are generally not provided any analgesic for pain relief, leading to welfare concerns. The objectives of this study were to assess the efficacy of two non-steroidal anti-inflammatory drugs (NSAIDs), meloxicam (MEL) (0.4 mg/kg or 1.0 mg/kg) and ketoprofen (KET) (6.0 mg/kg) in reducing behavioral indicators of pain in castrated piglets. This study also examined the utility of the Piglet Grimace Scale (PGS) as a pain assessment tool. Nineteen litters of 5-days-old male piglets (*n* = 120) were used and piglets within a litter were randomly assigned to one of eight possible treatments: 0.4 mg/kg MEL-castrated or uncastrated, 1.0 mg/kg MEL-castrated or uncastrated, 6.0 mg/kg KET-castrated or uncastrated, saline (castrated control), or sham (uncastrated control). Treatments were administered intramuscularly (IM) 20 min prior to surgical castration. Piglets were video recorded for 1 h pre-procedure, for 8 h immediately post-castration and for another hour, 24 h post-procedure. Twenty-one behaviors and postures were scored continuously for the first 15 min of each hour and 1,156 still images of piglet faces were collected and scored using the PGS. Within each treatment group post-castration, castrated piglets displayed significantly more pain-related behaviors than uncastrated piglets (0.4 mg/kg MEL: *p* = 0.0339, 1.0 mg/kg MEL: *p* = 0.0079, 6.0 mg/kg KET: *p* = 0.0034, Controls: *p* < 0.0001). Castrated piglets also grimaced significantly more post-procedure than uncastrated piglets (*p* = 0.0061). Compared to the castrated control, none of the NSAID treatments significantly reduced piglet pain behaviors (0.4 mg/kg MEL: *p* = 1.0000, 1.0 mg/kg MEL: *p* = 0.9995, 6.0 mg/kg KET: *p* = 0.4163) or facial grimacing. Piglets demonstrated significantly more pain behaviors 24 h post-castration than at all other time points (*p* < 0.0001). The PGS was a less effective measure to detect acute pain; however, our findings suggest it does have utility as a pain assessment tool in neonatal pigs. Our findings also indicate that the use of these NSAIDs were ineffective at alleviating castration-associated pain in piglets.

## Introduction

Piglets are surgically castrated on commercial pig production farms to prevent boar taint and reduce aggression (1). This is known to be a painful procedure, based on specific behavioral and physiologic indicators, including rump scratching, increased blood cortisol levels, and high-frequency vocalizations (2–4), yet piglets are generally not provided any analgesia or anesthesia for pain relief. This has been recognized as a significant welfare concern in pig production, with guidelines in the EU and Canada now requiring analgesia administration prior to surgical castration (5, 6). Non-steroidal anti-inflammatory drugs (NSAIDs), such as meloxicam (MEL) and ketoprofen (KET), are the most common type of analgesic given to food animals and are currently being recommended for use in piglets to alleviate pain. The label dose of meloxicam (0.4 mg/kg) has had variable success in significantly reducing surgical castration pain (4, 7). There is limited research on the use of ketoprofen for pain control in piglets following castration.

A Piglet Grimace Scale (PGS) was developed by our research group at the University of Guelph to rapidly assess pain based on piglet facial expressions (8). How well piglet grimacing corresponds to expression of pain behaviors is important to determine the accuracy of this novel pain assessment tool.

The objectives of this study were to assess the effectiveness of meloxicam at the label dose (0.4 mg/kg) and a high dose (1.0 mg/kg), as well as, ketoprofen (6.0 mg/kg) in reducing pain resulting from surgical castration of piglets. We hypothesized that piglets receiving 1.0 mg/kg meloxicam would show the greatest reduction in pain behaviors. This study also aimed to determine if the PGS could be used as a pain assessment tool by comparing it against castration-related pain behaviors.

## Materials and methods

All animal use and procedures were approved by the University of Guelph Animal Care Committee (Animal Utilization Protocol #3350). The institution is registered under the Animals for Research Act of Ontario and holds a Good Animal Practice certificate issued by the Canadian Council on Animal Care.

### Animals and treatments

A total of 120 Yorkshire-Landrace × Duroc male piglets (5-days-old, 1.15–2.95 kg BW) from 19 different litters were used in this study. Cross-fostering of piglets did occur on-farm when necessary, but litters of piglets were selected for this study that remained with their biological sow. Sows and piglets were housed in farrowing pens at the University of Guelph Arkell Swine Research Station. The floor space for each pen was 1.8 × 2.4 m and the farrowing crate was 0.8 × 2.3 m. The farrowing rooms were maintained at ambient temperature (23 ± 0.5°C) with lights on/off at 07:00/21:00, and natural light was provided by windows in each room. Sows were fed *ab libitum* beginning 4 days after farrowing. The creep areas for piglets were heated to approximately 30–35°C by means of a heating pad or lamp (the farrowing pens in this study were equipped with heat lamps).

Eight treatments were used, and each treatment group was identified by a unique symbol (a T, V, X, ∞, #, diamond, heart, or square) that was marked on the piglet's forehead and back with a black permanent marker prior to castration. This was to ensure that individuals involved in most aspects of the study (e.g., scoring behavior, pulling images of piglet faces, statistical analysis) were unaware of animal treatment. Marking the piglet's forehead was necessary to maintain animal-treatment group identification when only observing piglet faces. Numbers were also written on the back leg of piglets for individual identification. Fifteen piglets were assigned to each treatment group. Group size was based on a sample size estimate, using α = 0.05, population σ = 0.1 (determined from a pilot study) and 5% precision (8, 9). Within each litter, piglets were randomly assigned to one of the following treatments: 0.4 mg/kg meloxicam-castrated, 0.4 mg/kg meloxicam-uncastrated, 1.0 mg/kg meloxicam-castrated, 1.0 mg/kg meloxicam-uncastrated, 6.0 mg/kg ketoprofen-castrated, 6.0 mg/kg ketoprofen-uncastrated, saline (castrated control), or sham (uncastrated control). Meloxicam (MEL) (Metacam 20 mg/mL; Boehringer Ingelheim Ltd., Burlington, ON, Canada) was administered as an intramuscular (IM) injection at the label dose (0.4 mg/kg) and a higher, semi-log increment dose (1.0 mg/kg). Metacam was diluted to 4 mg/mL for administration of 0.4 mg/kg and 10 mg/mL for administration of 1.0 mg/kg, to give an average volume of ~0.2 mL to all piglets (range: 0.1–0.3 mL/piglet). Ketoprofen (KET) (Anafen 100 mg/mL; Merial Canada Inc., Baie-D'Urfé, QC, Canada; extra-label use) was administered IM and diluted to 80 mg/mL to administer ~0.2 mL to all piglets (range: 0.1–0.2 mL/piglet). Ketoprofen dose was derived from the literature (10). Saline was given IM at 0.2 mL/piglet. The sham treatment group was only handled and did not receive an injection.

### Processing procedures

Twenty-four hours prior to the trial, piglets were weighed and marked with the symbol that corresponded to their treatment group (treatments were not piglet weight-balanced; mean piglet weight in each treatment group is presented in **Table 2**). On the day of castration, male piglets within a pen were separated from their littermates, placed in a transport cart, and administered their assigned treatments 20 min prior to castration. Piglets were surgically castrated using two vertical incisions and tearing of the spermatic cord (11) and then returned to their pen. Separation from the sow and littermates lasted ~30–40 min. All castrations occurred between 08:00 and 10:00 and were conducted by one individual (AVV). Piglets in the sham treatment group were positioned on the leg of the castrator as if they were about to undergo the procedure, the handle of the scalpel was used to simulate the incision and the scrotum was manipulated to resemble a surgical castration. Uncastrated-treatment piglets were given an IM injection only and did not undergo a simulated castration.

### Behavioral recording and scoring

Piglets were video recorded pre-procedure for 1 h using a high definition video camera (JVC GZ-E200 full HD Everio Camcorder, Yokohama, Japan) mounted on a tripod outside of the farrowing pens. Immediately post-castration, piglets were video recorded continuously for 8 h, and again for 1 h at 24 h post-procedure (i.e., 10 h of video data were collected in total for each litter of pigs). The behavior of each piglet was scored continuously by four trained observers for the first 15 min of every hour of data collected using the Observer XT program (Version 12.0: Noldus Information Technology, Wageningen, The Netherlands) according to a detailed ethogram adapted from Hay et al. (2) (Table 1). The observers were blinded as to time point, litter, and piglet treatment; however, they could observe which piglets had been castrated and which had not. Two observers scored two pens each, one observer scored six pens, and one observer scored nine pens. The scoring reliability between the four observers was assessed at three points during the behavior scoring period, by having all participants score the same piglet in a video. The intraclass correlation coefficient (ICC) was calculated to ensure behaviors were being scored consistently (all interobserver reliability tests produced an ICC above 0.9, indicating excellent agreement between scorers). A total of 18,000 min (300 h) of behavior recordings were scored and analyzed for this study (2.5 h per piglet).

**Table 1:** Ethogram used to score piglet behavior, grouped into feeding, locomotion, non-specific behaviors, castration-related pain behaviors, posture, and social cohesion [adapted from Hay et al. (2)].

**Behaviors**	**Description**
Suckling^a^	Teat in mouth and suckling movements
Nosing udder^a^	Nose in contact with udder, up and down head movements
Playing^a^	Springing (sudden jumping or leaping), head shaking, horizontal, or vertical bouncy movements with or without littermates
Agonistic^a^	Biting or fighting other littermates
Walking^a^	Moving forward at a normal pace
Running^a^	Moving forward at a rapid pace (trot or gallop)
Awake inactive^b^	No special activity, but awake
Sleeping^b^	Lying down, eyes closed
Nosing^a^	Snout in contact with a substrate
Chewing^a^	Nibbling at littermates or substrates
Trembling^c^	Shivering, as with cold
Spasms^c^	Quick and involuntary contractions of the muscles
Scratching^c^	Rubbing the rump against the floor, pen walls, or littermates
Tail wagging^c^	Tail's movements from side to side (or up and down)
Stiffness^c^	Lying with extended and tensed legs
Lying^b^	Body weight supported by side or belly
Sitting^a^	Body weight supported by hindquarters and front legs
Standing^a^	Body weight supported by four legs
Kneeling^a^	Body weight supported by front carpal joints and hind legs
Isolated^a, b^	Alone or with one littermate at most, distance of 40 cm separates the animal(s) from the closest group of littermates
Desynchronized^a^^,^ ^b^	Activity different from that of most littermates (at least 75%)

a *Active behaviors*.

b *Inactive behaviors*.

c *Castration-related pain behaviors*.

Piglet behaviors were analyzed individually and then grouped into “active,” “inactive,” and “pain” categories, to assess the activity level of piglets across the observation period and the total proportion of pain behaviors displayed. Active behaviors included playing, walking, suckling, nosing, chewing, and running. Inactive behaviors included awake inactive and sleeping. Postures were used for this behavioral analysis; piglets that were sitting or standing were scored as demonstrating an “active” behavior and piglets that were lying were scored as exhibiting an “inactive” behavior. Sitting was placed in the active category because most piglets assumed this posture when suckling or scratching the rump and these were considered active behaviors. Pain behaviors included trembling, stiffness, spasms, tail wagging, and rump scratching (2).

### Piglet grimace scale (PGS) recording and scoring

Still images of piglet faces were captured from the first 30 min of every hour of video data collected by an individual blinded as to animal treatment and time point. Videos were uploaded to the Everio MediaBrowser 4 program (Pixela Corporation, Osaka, Japan) and whenever a piglet face was in view, the video was paused, and the still image was captured (excluding times when piglets were lying with their head down or sleeping). Taking at least one facial image of each piglet per time point in this study was attempted. A total of 1,156 facial images were captured (Table 2). Prior to scoring, the images were uploaded to Photoshop (Adobe Systems Incorporated, San Jose, CA) and the symbol marked on each piglet's forehead was blurred to ensure volunteer scorers were blind to treatment. Faces were then randomized into files using a random number generator (random.org).

**Table 2:** Total number of piglet faces captured for Piglet Grimace Scale scoring.

**Time point (h)**	**Treatment**								**Total**
	**0.4 mg/kg MEL cast (2.28 ± 0.2 kg)^a^**	**0.4 mg/kg MEL uncast (2.36 ± 0.1 kg)**	**1.0 mg/kg MEL cast (2.28 ± 0.2 kg)**	**1.0 mg/kg MEL uncast (2.22 ± 0.1 kg)**	**6.0 mg/kg KET cast (2.28 ± 0.1 kg)**	**6.0 mg/kg KET uncast (2.31 ± 0.1 kg)**	**Saline (2.28 ± 0.0 kg)**	**Sham (2.03 ± 0.1 kg)**
pre	18	15	22	15	20	12	16	9	127
0	28	14	13	17	23	14	25	16	150
1	18	12	21	24	14	15	15	14	133
2	26	10	15	8	11	9	16	13	108
3	18	17	12	10	11	13	13	11	105
4	21	9	13	11	15	12	10	10	101
5	18	10	12	7	15	8	15	8	93
6	24	16	8	7	16	11	13	10	105
7	13	12	13	15	4	9	10	4	80
24	29	16	16	21	15	13	24	20	154
Total	213	131	145	135	144	116	157	115	1,156

a *Mean weight of piglets ± SE (n = 15) in each treatment group. There was no statistically significant difference in weight between any of the treatment groups*.

**Figure 1:**
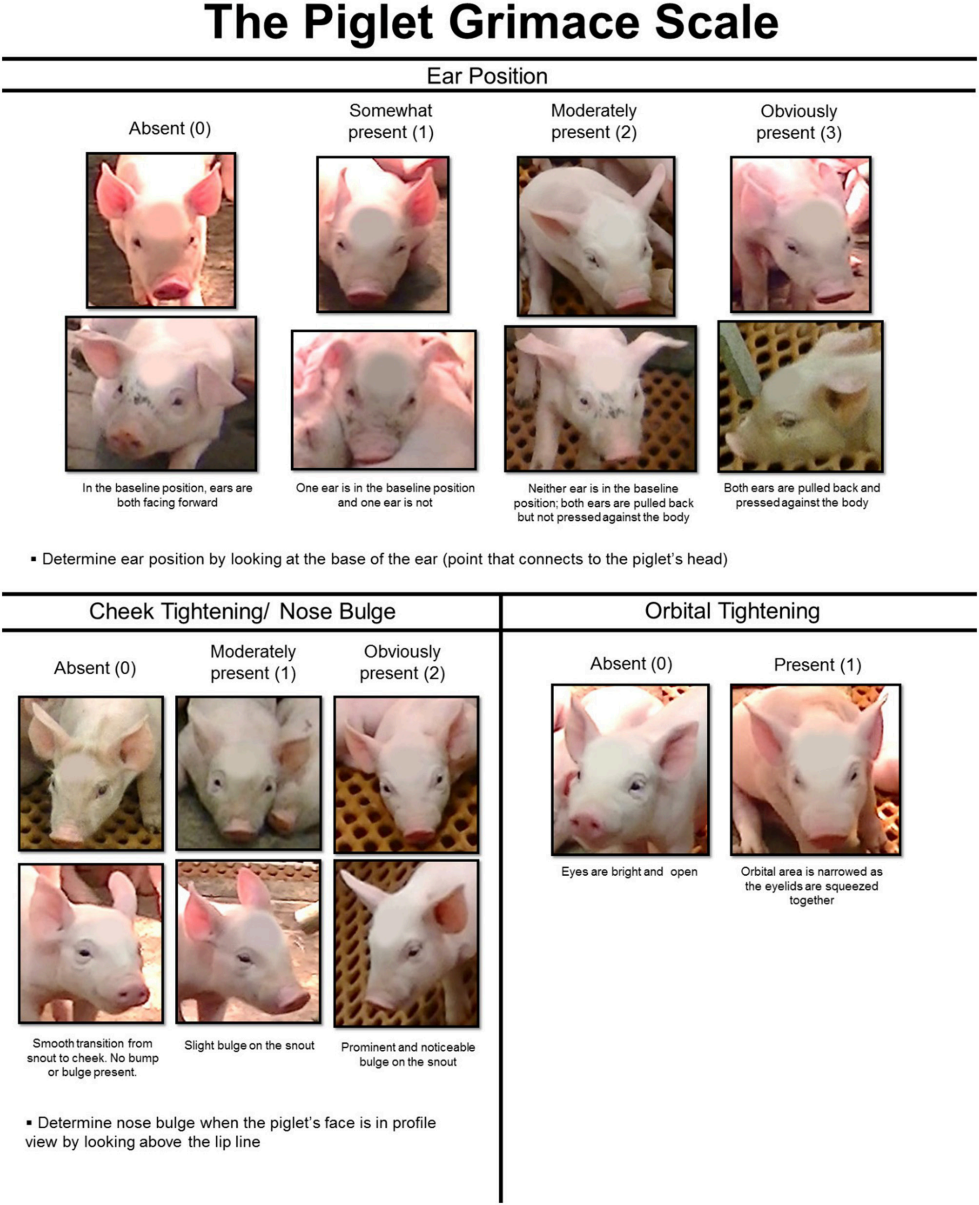
The Piglet Grimace Scale (PGS) is based on scoring three facial action units (FAUs): ear position, cheek tightening/nose bulge, and orbital tightening.

The preliminary PGS (8) was modified for this study (Figure 1). Ear position, which was originally placed on a 3-point scale (0–2), was expanded to a 4-point scale (0–3). Images of piglets with upright and floppy ears were included to make scoring ear position easier. Both front-facing piglets and profile images were added to the cheek tightening/nose bulge category and descriptive text was provided to explain the facial feature changes in detail. The maximum pain score using the revised PGS was 6. These changes were made to make the PGS more sensitive to pain expression, allowing for better reliability and to make the scale easier to use.

Eight individuals blinded as to piglet treatment, litter, and time point used the PGS to score each image. If an image could not be scored reliably, for example, due to poor image quality, the volunteers were instructed to exclude it from scoring (15 images were removed in total because of reported quality issues). The PGS score for each image was calculated by summing the scores given to the facial action units (ear position, cheek tightening/nose bulge, and orbital tightening). If more than one image was pulled for a piglet at the same time point, the PGS scores were averaged across images prior to analysis, to prevent pseudo-replication. The final PGS score of each piglet per time point was calculated as a mean of the scores from the eight individuals.

### Data and statistical analysis

The total duration of behaviors was converted into proportion of time a piglet engaged in each behavior prior to analysis (to account for periods of time when the piglet was not in view and could not be scored). Normality was evaluated using the univariate procedure in SAS (Statistical Analysis System 9.4, SAS Institute Inc., NC). Data were analyzed using a GLIMMIX procedure with a beta distribution, including treatment, time, litter, and time x treatment interaction. Litter was included as a random effect and time was a repeated measure with piglet as the experimental unit. *Post hoc* tests were conducted using the Tukey-Kramer adjustment, to control the false-positive rate (i.e., incidence of Type I error) for multiple comparisons (12). Statistical significance was set at *p* < 0.05.

The grimace scale scores were analyzed using a mixed procedure, including litter, time, treatment, and time x treatment interaction. Litter was included as a random effect and time was a repeated measure with piglet as the experimental unit. A *post hoc* Tukey's test was conducted for significant outcomes.

The treatment variable was first set as each NSAID and control treatment administered to the piglets. When no significant effect of treatment and treatment × time interaction were found on any behavioral variable between 0.4 mg/kg MEL-castrated, 1.0 mg/kg MEL-castrated, 6.0 mg/kg KET-castrated, and saline piglets, data was pooled into a “castrated” group for further analysis. Similarly, when no significant effect of treatment and time x treatment interaction were found on any behavioral variable between 0.4 mg/kg MEL-uncastrated, 1.0 mg/kg MEL-uncastrated, 6.0 mg/kg KET-uncastrated, and sham piglets, data was pooled into a “uncastrated” group. These castrated and uncastrated groups were assessed for treatment and time × treatment effects. A final analysis was conducted on NSAID-castrated (0.4 mg/kg MEL-castrated, 1.0 mg/kg MEL-castrated, and 6.0 mg/kg KET-castrated) and NSAID-uncastrated (0.4 mg/kg MEL-uncastrated, 1.0 mg/kg MEL-uncastrated, and 6.0 mg/kg KET-uncastrated) piglet groups. Both behavioral and PGS data were used to assess the effectiveness of NSAID treatment in reducing surgical castration pain.

## Results

### Behavioral observations

#### Comparison between NSAID-treated and control piglets

Nine individual behaviors were significantly affected by time across the observation period: awake inactive (*p* < 0.0001), lying (*p* < 0.0001), nosing (*p* < 0.0001), sleeping (*p* < 0.0001), standing (*p* < 0.0001), suckling (*p* < 0.0001), tail wagging (*p* < 0.0001), walking (*p* < 0.0001), and chewing (*p* = 0.0129) (Table 3). Across all treatment groups, piglets spent significantly more time walking and standing and less time lying at 0 and 24 h post-castration compared to all other time points (*p* < 0.05). At 24 h post-castration, they spent significantly more time nosing and wagging their tails (*p* < 0.05). At 0 h post-castration, all piglets spent significantly less time sleeping and were more awake inactive than at all other time points, except at 24 h (*p* < 0.05). Compared to pre-castration and 24 h post-castration, piglets slept significantly more at 7 h (*p* < 0.05). All piglets spent significantly more time suckling 5 h post-castration than at all other time points, except at 3 and 7 h (*p* < 0.05). Piglets demonstrated more chewing behaviors at 5 h post-castration than pre-castration (*p* = 0.0186). There were no significant behavioral differences between any of the treatment groups pre-castration (*p* > 0.05).

**Table 3:** Behavioral analysis of piglets (*n* = 120) across all litters, replicates and time points.

				**Time (post-castration)**									
	**Behavior**	***F*-value**	**Pr^1^ > F**	**Pre (115)^2^**	**0 h (120)**	**1 h (117)**	**2 h (120)**	**3 h (120)**	**4 h (119)**	**5 h (118)**	**6 h (119)**	**7 h (119)**	**24 h (118)**
Proportion of time (duration)	Awake inactive	7.09	<**0.0001**	0.54 ± 0.03^ac^	0.62 ± 0.03^a^	0.47 ± 0.03^bc^	0.47 ± 0.03^bc^	0.50 ± 0.03^bc^	0.47 ± 0.03^bc^	0.60 ± 0.03^a^	0.45 ± 0.03^bc^	0.46 ± 0.03^bc^	0.53 ± 0.03^ac^
	Lying	11.79	<**0.0001**	0.66 ± 0.03^b^	0.45 ± 0.03^a^	0.68 ± 0.03^b^	0.67 ± 0.03^b^	0.70 ± 0.03^b^	0.75 ± 0.03^b^	0.67 ± 0.03^b^	0.71 ± 0.03^b^	0.71 ± 0.03^b^	0.55 ± 0.03^a^
	Nosing	6.59	<**0.0001**	0.05 ± 0.01^ac^	0.04 ± 0.00^a^	0.03 ± 0.00^a^	0.03 ± 0.00^a^	0.03 ± 0.00^a^	0.03 ± 0.00^a^	0.03 ± 0.00^a^	0.03 ± 0.00^a^	0.03 ± 0.00^a^	0.07 ± 0.01^bc^
	Nosing udder	1.04	0.4077	0.16 ± 0.05	0.27 ± 0.05	0.20 ± 0.05	0.21 ± 0.05	0.21 ± 0.05	0.17 ± 0.04	0.27 ± 0.06	0.15 ± 0.04	0.16 ± 0.04	0.14 ± 0.05
	Sleeping	6.56	<**0.0001**	0.40 ± 0.04^ac^	0.34 ± 0.04^a^	0.50 ± 0.04^bc^	0.52 ± 0.04^bc^	0.48 ± 0.04^bc^	0.54 ± 0.04^bd^	0.44 ± 0.04^acd^	0.54 ± 0.04^bd^	0.57 ± 0.04^b^	0.43 ± 0.04^acd^
	Standing	13.15	<**0.0001**	0.29 ± 0.03^b^	0.52 ± 0.04^a^	0.28 ± 0.03^b^	0.30 ± 0.03^b^	0.27 ± 0.03^b^	0.22 ± 0.03^b^	0.27 ± 0.03^b^	0.25 ± 0.03^b^	0.25 ± 0.03^b^	0.41 ± 0.04^a^
	Suckling	5.96	<**0.0001**	0.16 ± 0.02^ad^	0.18 ± 0.02^ad^	0.16 ± 0.02^ad^	0.14 ± 0.02^a^	0.23 ± 0.03^cd^	0.18 ± 0.03^ad^	0.28 ± 0.03^bc^	0.18 ± 0.03^ad^	0.21 ± 0.03^ac^	0.11 ± 0.02^a^
	Tail wagging	14.93	<**0.0001**	0.02 ± 0.00^a^	0.03 ± 0.00^a^	0.03 ± 0.00^a^	0.04 ± 0.00^a^	0.04 ± 0.00^a^	0.02 ± 0.00^a^	0.02 ± 0.00^a^	0.03 ± 0.00^a^	0.03 ± 0.00^a^	0.09 ± 0.01^b^
	Walking	24.25	<**0.0001**	0.09 ± 0.01^b^	0.17 ± 0.02^a^	0.08 ± 0.01^bc^	0.10 ± 0.01^b^	0.07 ± 0.01^bc^	0.05 ± 0.00^c^	0.04 ± 0.00^c^	0.06 ± 0.01^bc^	0.06 ± 0.00^c^	0.16 ± 0.02^a^
	Sitting	1.08	0.3767	0.05 ± 0.00	0.04 ± 0.00	0.06 ± 0.01	0.05 ± 0.00	0.05 ± 0.00	0.06 ± 0.01	0.07 ± 0.01	0.06 ± 0.01	0.06 ± 0.01	0.06 ± 0.00
	Spasms	0.72	0.6927	0.00 ± 0.00	0.00 ± 0.00	0.00 ± 0.00	0.00 ± 0.00	0.00 ± 0.00	0.00 ± 0.00	0.00 ± 0.00	0.00 ± 0.00	0.01 ± 0.00	0.00 ± 0.00
	Kneeling	1.17	0.3175	0.01 ± 0.00	0.02 ± 0.00	0.01 ± 0.00	0.00 ± 0.00	0.01 ± 0.00	0.00 ± 0.00	0.02 ± 0.00	0.01 ± 0.00	0.01 ± 0.00	0.01 ± 0.00
	Playing	1.50	0.1540	0.02 ± 0.00	0.04 ± 0.01	0.00 ± 0.00	0.05 ± 0.01	0.04 ± 0.01	0.01 ± 0.00	0.00 ± 0.00	0.04 ± 0.01	0.03 ± 0.01	0.04 ± 0.00
	Scratching	2.53	**0.0103**^3^	0.00 ± 0.00	0.00 ± 0.00	0.01 ± 0.00	0.00 ± 0.00	0.00 ± 0.00	0.00 ± 0.00	0.00 ± 0.00	0.00 ± 0.00	0.00 ± 0.00	0.02 ± 0.00
	Isolated	1.40	0.1952	0.11 ± 0.04	0.19 ± 0.06	0.10 ± 0.04	0.13 ± 0.04	0.08 ± 0.04	0.06 ± 0.03	0.11 ± 0.03	0.08 ± 0.04	0.08 ± 0.03	0.06 ± 0.03
	Desynchronized	1.73	0.0840	0.12 ± 0.05	0.15 ± 0.05	0.12 ± 0.04	0.05 ± 0.02	0.06 ± 0.03	0.06 ± 0.03	0.10 ± 0.04	0.13 ± 0.05	0.12 ± 0.05	0.11 ± 0.05
	Stiffness	1.54	0.1462	0.00 ± 0.00	–	0.00 ± 0.00	0.01 ± 0.00	0.00 ± 0.00	0.00 ± 0.00	0.00 ± 0.00	0.00 ± 0.00	0.01 ± 0.00	0.01 ± 0.00
	Chewing	2.46	**0.0129**	0.00 ± 0.00^a^	0.01 ± 0.00^ab^	0.00 ± 0.00^ab^	0.01 ± 0.00^ab^	0.02 ± 0.00^ab^	0.00 ± 0.00^ab^	0.02 ± 0.00^b^	0.01 ± 0.00^ab^	0.01 ± 0.00^ab^	0.01 ± 0.00^ab^
	Trembling	0.06	0.9998	0.01 ± 0.03	0.00 ± 0.03	0.02 ± 0.04	0.07 ± 0.36	0.01 ± 0.03	0.03 ± 0.04	0.06 ± 0.73	0.00 ± 0.02	0.03 ± 0.06	–
	Running	0.05	1.0000	0.01 ± 0.04	0.00 ± 0.02	0.02 ± 0.07	0.00 ± 0.03	0.01 ± 0.05	0.00 ± 0.02	0.00 ± 0.03	0.02 ± 0.05	0.03 ± 0.09	0.00 ± 0.01
	Agonistic	1.41	0.1999	–^6^	–	–	–	–	–	–	–	–	–
	Active^4^	11.24	<**0.0001**	0.33 ± 0.03^b^	0.56 ± 0.03^a^	0.32 ± 0.03^b^	0.32 ± 0.03^b^	0.29 ± 0.03^b^	0.25 ± 0.03^b^	0.32 ± 0.03^b^	0.30 ± 0.03^b^	0.28 ± 0.03^b^	0.46 ± 0.03^a^
	Pain^5^	8.51	<**0.0001**	0.03 ± 0.00^a^	0.05 ± 0.00^a^	0.05 ± 0.00^a^	0.06 ± 0.01^a^	0.05 ± 0.00^a^	0.04 ± 0.00^a^	0.04 ± 0.00^a^	0.04 ± 0.00^a^	0.05 ± 0.00^a^	0.11 ± 0.01^b^

*Values presented are the proportional means ± SE*.

a−d *Means with different superscripts in the same row differ significantly (p < 0.05)*.

1 *Significant effects are indicated in bold*.

2 *Total number of observations for each treatment group*.

3 *Not significant after Tukey-Kramer adjustment*.

4 *Active behaviors include: nosing, suckling, walking, chewing, playing, and running*.

5 *Pain behaviors include: stiffness, trembling, spasms, tail wagging, and scratching*.

6 *Dash indicates behavior was not observed*.

**Figure 2:**
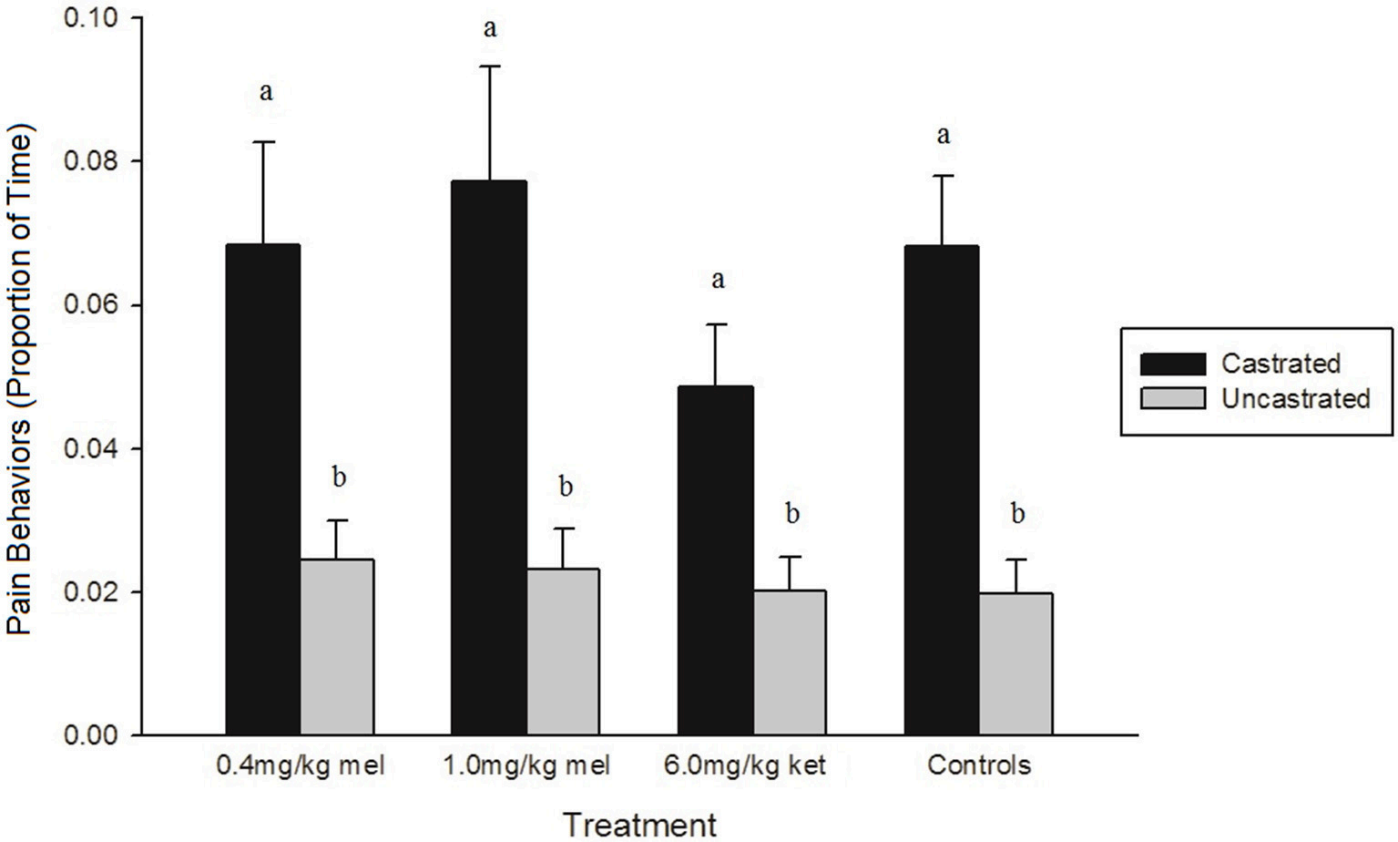
The proportion of time (± SE) piglets demonstrated pain-related behaviors (trembling, stiffness, spasms, tail wagging, and rump scratching) in each treatment group. The control groups were saline-castrated and sham-uncastrated piglets (*n* = 15 piglets/treatment group). Observers (*n* = 4) were unaware of piglet treatment, litter, and time point when scoring. Different letters (a, b) indicate significant differences between treatments (*p* < 0.05).

Only three individual behaviors, tail wagging (*p* < 0.0001), walking (*p* = 0.0042), and kneeling (*p* = 0.0261), were affected by treatment across all time points (Table 4). Within each treatment group, castrated piglets wagged their tails significantly more than uncastrated piglets (0.4 mg/kg MEL: *p* = 0.0432, 1.0 mg/kg MEL: *p* = 0.0228, 6.0 mg/kg KET: *p* = 0.0098, Controls: *p* < 0.0001). Piglets in the 0.4 mg/kg MEL-castrated group walked significantly less than piglets in the 1.0 mg/kg MEL-castrated group (*p* = 0.0095). Piglets in the 0.4 mg/kg MEL-uncastrated group spent significantly more time kneeling than piglets in the 1.0 mg/kg MEL-uncastrated group (*p* = 0.0235). Castrated piglets also displayed significantly more pain behaviors than uncastrated piglets within each treatment group (0.4 mg/kg MEL: *p* = 0.0339, 1.0 mg/kg MEL: *p* = 0.0079, 6.0 mg/kg KET: *p* = 0.0034, Controls: *p* < 0.0001) (Figure 2). None of the NSAID treatment groups significantly reduced piglet pain behaviors post-castration (0.4 mg/kg MEL: *p* = 1.0000, 1.0 mg/kg MEL: *p* = 0.9995, 6.0 mg/kg KET: *p* = 0.4163).

**Table 4:** Behavioral analysis of piglets (*n* = 120) across all litters, replicates and time points.

				**Treatment**							
	**Behavior**	***F*-value**	**Pr^1^ > F**	**0.4 mg/kg MEL cast (155)^2^**	**0.4 mg/kg MEL uncast (150)**	**1.0 mg/kg MEL cast (145)**	**1.0 mg/kg MEL uncast (150)**	**6.0 mg/kg KET cast (146)**	**6.0 mg/kg KET uncast (150)**	**Saline (142)**	**Sham (147)**
Proportion of time (duration)	Awake inactive	0.29	0.9589	0.48 ± 0.05	0.53 ± 0.04	0.49 ± 0.05	0.50 ± 0.04	0.51 ± 0.04	0.53 ± 0.04	0.53 ± 0.03	0.53 ± 0.04
	Lying	0.38	0.9129	0.59 ± 0.06	0.68 ± 0.04	0.63 ± 0.06	0.66 ± 0.04	0.66 ± 0.04	0.67 ± 0.04	0.68 ± 0.03	0.69 ± 0.04
	Nosing	1.96	0.0585	0.02 ± 0.00	0.05 ± 0.01	0.03 ± 0.00	0.04 ± 0.01	0.04 ± 0.01	0.04 ± 0.01	0.04 ± 0.00	0.03 ± 0.00
	Nosing udder	0.13	0.9964	0.27 ± 0.12	0.16 ± 0.06	0.25 ± 0.12	0.15 ± 0.05	0.19 ± 0.06	0.16 ± 0.06	0.19 ± 0.05	0.19 ± 0.06
	Sleeping	0.83	0.5661	0.56 ± 0.06	0.41 ± 0.04	0.55 ± 0.06	0.44 ± 0.05	0.49 ± 0.04	0.44 ± 0.05	0.46 ± 0.04	0.45 ± 0.04
	Standing	0.38	0.9119	0.36 ± 0.06	0.29 ± 0.04	0.36 ± 0.06	0.28 ± 0.04	0.30 ± 0.04	0.28 ± 0.04	0.28 ± 0.03	0.26 ± 0.04
	Suckling	0.89	0.5127	0.18 ± 0.04	0.17 ± 0.03	0.21 ± 0.04	0.16 ± 0.03	0.20 ± 0.03	0.19 ± 0.03	0.16 ± 0.02	0.17 ± 0.03
	Tail wagging	7.15	<**0.0001**	0.06 ± 0.01^a^	0.02 ± 0.00^bc^	0.07 ± 0.01^a^	0.02 ± 0.00^bc^	0.04 ± 0.00^ac^	0.02 ± 0.00^b^	0.06 ± 0.01^a^	0.01 ± 0.00^b^
	Walking	2.99	**0.0042**	0.06 ± 0.01^a^	0.10 ± 0.01^ab^	0.09 ± 0.02^b^	0.09 ± 0.02^ab^	0.08 ± 0.01^ab^	0.10 ± 0.02^ab^	0.07 ± 0.01^ab^	0.07 ± 0.01^ab^
	Sitting	0.97	0.4532	0.07 ± 0.02	0.06 ± 0.01	0.05 ± 0.02	0.06 ± 0.01	0.05 ± 0.01	0.05 ± 0.01	0.05 ± 0.01	0.06 ± 0.01
	Spasms	0.54	0.8040	0.01 ± 0.00	0.00 ± 0.00	0.01 ± 0.00	0.00 ± 0.00	0.00 ± 0.00	0.00 ± 0.00	0.00 ± 0.00	0.00 ± 0.00
	Kneeling	2.32	**0.0261**	0.02 ± 0.00^ab^	0.01 ± 0.00^a^	0.02 ± 0.00^ab^	0.00 ± 0.00^b^	0.01 ± 0.00^ab^	0.01 ± 0.00^ab^	0.01 ± 0.00^ab^	0.00 ± 0.00^ab^
	Playing	1.52	0.1669	0.03 ± 0.03	0.02 ± 0.01	0.05 ± 0.03	0.02 ± 0.01	0.00 ± 0.00	0.02 ± 0.01	0.02 ± 0.01	0.00 ± 0.00
	Scratching	0.94	0.4813	0.00 ± 0.01	0.01 ± 0.00	0.00 ± 0.00	0.00 ± 0.00	0.01 ± 0.00	0.00 ± 0.00	0.01 ± 0.00	0.00 ± 0.00
	Isolated	1.08	0.3794	0.04 ± 0.03	0.13 ± 0.06	0.03 ± 0.02	0.12 ± 0.05	0.19 ± 0.07	0.09 ± 0.05	0.09 ± 0.04	0.18 ± 0.07
	Desynchronized	1.69	0.1146	0.13 ± 0.12	0.09 ± 0.05	0.05 ± 0.07	0.12 ± 0.06	0.17 ± 0.07	0.07 ± 0.04	0.09 ± 0.04	0.09 ± 0.05
	Stiffness	0.98	0.4449	0.01 ± 0.00	0.00 ± 0.00	0.02 ± 0.00	0.00 ± 0.00	0.00 ± 0.00	0.00 ± 0.00	0.00 ± 0.00	0.00 ± 0.00
	Chewing	1.20	0.3100	0.00 ± 0.00	0.02 ± 0.00	0.00 ± 0.00	0.02 ± 0.00	0.02 ± 0.00	0.02 ± 0.00	0.00 ± 0.00	0.02 ± 0.00
	Trembling	0.07	0.9991	0.02 ± 0.08	0.04 ± 0.35	0.04 ± 0.19	0.08 ± 0.62	0.00 ± 0.02	0.02 ± 0.18	0.02 ± 0.05	0.00 ± 0.02
	Running	0.02	1.0000	0.00 ± 0.06	0.00 ± 0.04	0.00 ± 0.03	0.01 ± 0.07	0.00 ± 0.03	0.01 ± 0.08	0.01 ± 0.07	0.00 ± 0.08
	Agonistic	1.51	0.1883	–^5^	–	–	–	–	–	–	–
	Active^3^	0.47	0.8557	0.41 ± 0.06	0.32 ± 0.04	0.36 ± 0.06	0.34 ± 0.04	0.34 ± 0.04	0.32 ± 0.04	0.32 ± 0.03	0.31 ± 0.04
	Pain^4^	7.97	<**0.0001**	0.07 ± 0.01^a^	0.02 ± 0.00^b^	0.08 ± 0.02^a^	0.02 ± 0.00^b^	0.05 ± 0.00^a^	0.02 ± 0.00^b^	0.07 ± 0.01^a^	0.02 ± 0.00^b^

*Values presented are the proportional means ± SE*.

a−c *Means with different superscripts in the same row differ significantly (p < 0.05)*.

1 *Significant effects are indicated in bold*.

2 *Total number of observations for each treatment group*.

3 *Active behaviors include: nosing, suckling, walking, chewing, playing, and running*.

4 *Pain behaviors include: stiffness, trembling, spasms, tail wagging and rump scratching*.

5 *Dash indicates behavior was not observed*.

**Figure 3:**
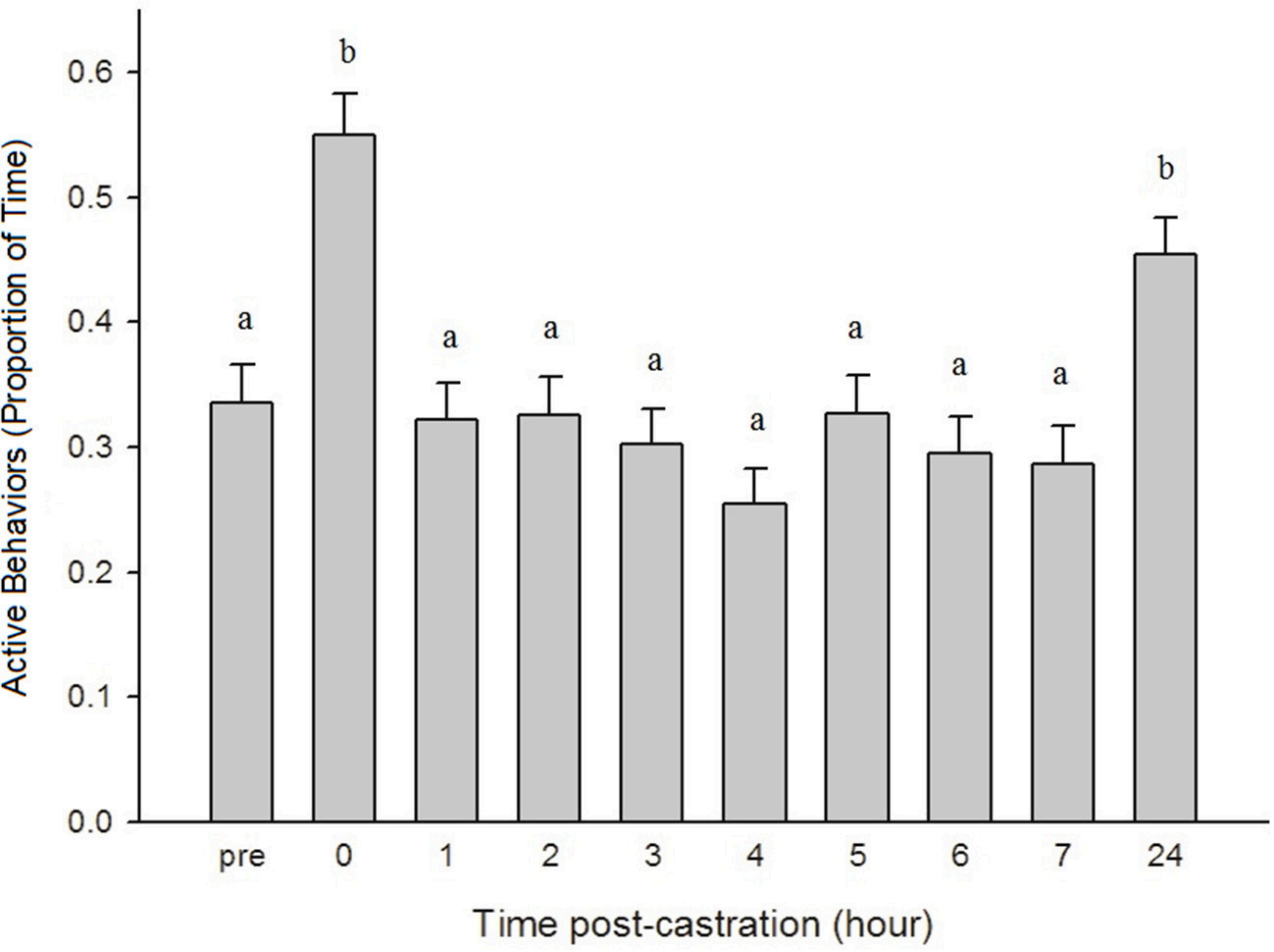
The proportion of time (± SE) piglets engaged in active behaviors (playing, walking, suckling, nosing, chewing, and running) throughout the observation period (*n* = 120 piglets/time point). Observers (*n* = 4) were unaware of piglet treatment, litter, and time point when scoring. Different letters (a, b) indicate significant differences between time points (*p* < 0.05).

There was no significant effect of treatment on piglet activity level (*p* = 0.8557) but there was a significant time effect, with piglets being more active at 0 h and 24 h post-castration compared to all other time points (*p* < 0.0001) (Figure 3).

#### Comparison between castrated and uncastrated piglets

**Figure 4:**
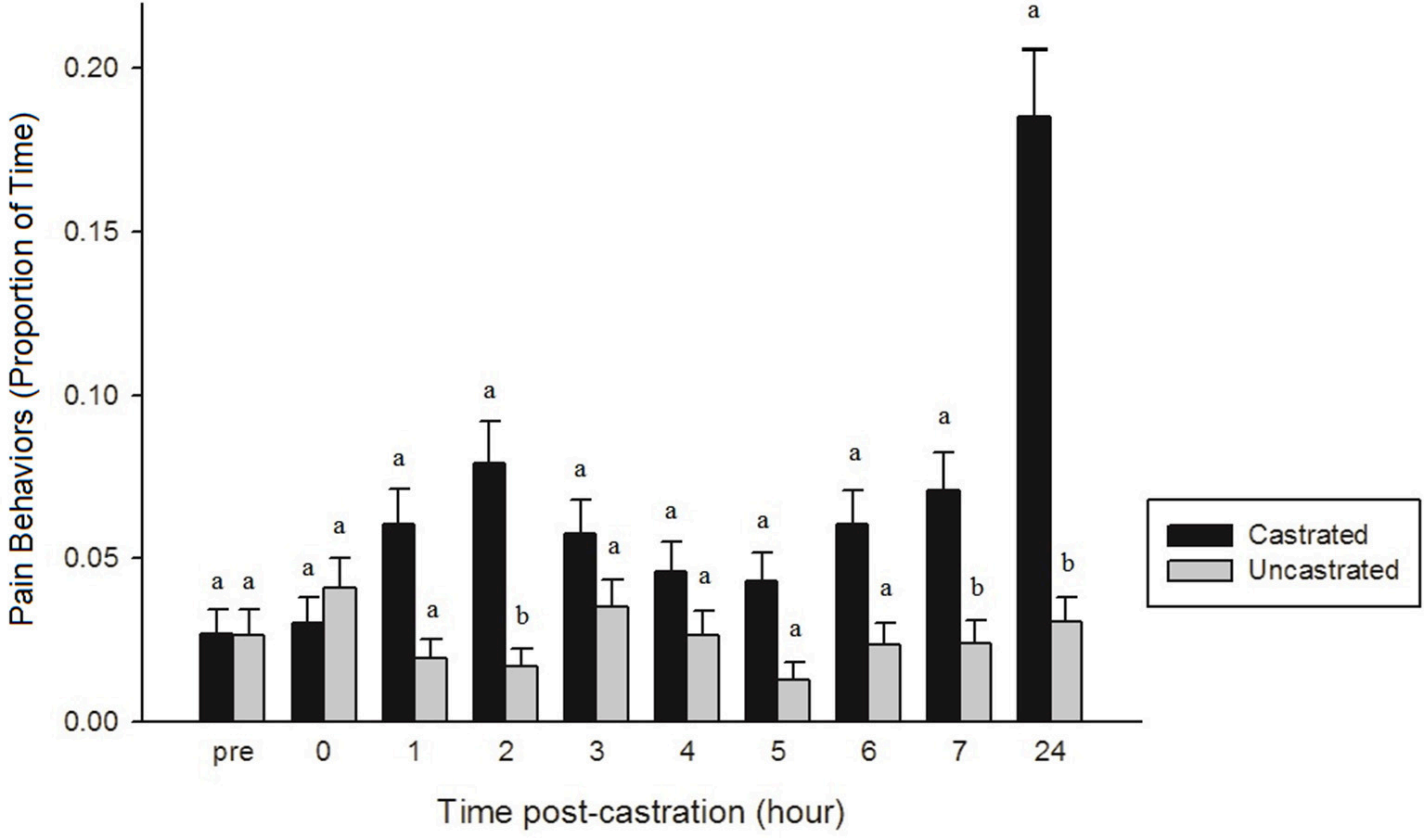
The proportion of time (± SE) castrated and uncastrated piglets demonstrated pain-related behaviors (trembling, stiffness, spasms, tail wagging, and rump scratching) throughout the observation period (*n* = 60 piglets/group). Observers (*n* = 4) were unaware of piglet treatment and time point when scoring. Different letters (a, b) indicate significant differences between groups within each time point (*p* < 0.05).

After analyzing the effect of each NSAID treatment on behavior and identifying no significant treatment-related effects, data were pooled into two groups: piglets that were castrated and those that were uncastrated. Only two behaviors, tail wagging (*p* < 0.0001) and pain (*p* < 0.0001), were significant across the entire observation period, with castrated piglets displaying significantly more tail wagging and pain-related behaviors than uncastrated piglets. There were also time x treatment interactions found for lying (*p* = 0.0027), sleeping (*p* = 0.0037), standing (*p* = 0.0024), tail wagging (*p* < 0.0001), walking (*p* < 0.0001), isolated (*p* = 0.0018), activity (*p* = 0.0045), and pain (*p* < 0.0001). At 0 h, castrated piglets spent significantly less time lying and more time standing, walking, and engaged in active behaviors than castrated piglets at 3, 4, and 7 h, and uncastrated piglets at 4 h (*p* < 0.05). At 0 h, uncastrated piglets were also significantly more active, spending more time standing and walking and less time lying and sleeping than both castrated and uncastrated piglets from 1 to 7 h post-castration (*p* < 0.05). At 24 h post-procedure, uncastrated piglets were significantly more active, spending less time lying and more time standing and walking than castrated piglets at 3, 4, and 7 h, and uncastrated piglets at 4 and 6 h (*p* < 0.05). Castrated piglets spent significantly more time isolated from their littermates at 0 h than castrated pigs from 2 to 5 h, 7, and 24 h and uncastrated piglets from 0, 1, 3 h to 24 h post-procedure (*p* < 0.05). Castrated piglets at 0–3 h, 6 and 7 h demonstrated significantly more tail wagging and pain-related behaviors than uncastrated piglets at 2 and 5 h (*p* < 0.05). At 24 h post-castration, castrated piglets were observed engaging in significantly more tail wagging and pain-related behaviors than both castrated and uncastrated piglets from 0 to 7 h (*p* < 0.0001) (Figure 4).

Data were also collapsed into two groups: NSAID-castrated piglets and NSAID-uncastrated piglets (after no behavior variables were found to be significant). The pre-treatment time point and the control piglets were removed from this analysis. The results were very similar to the above comparison between castrated and uncastrated piglets (Table 5).

**Table 5:** Behavioral analysis of piglets (*n* = 120) pre-treatment and post-treatment across all litters, replicates, and time points.

		**Pre-castration**		**Post-castration**				
	**Behavior^1^**	**Treatment *P*-value**	**Pre-treatment (115)^2^**	**Treatment *P*-value^3^**	**Time *P*-value**	Time*Treatment *P*-value	**NSAID-castrated^5^ (402)**	**NSAID-uncastrated^6^ (405)**
Proportion (Duration)	Awake inactive	0.8992	0.55 ± 0.05	0.6807	<**0.0001**	0.0581	0.50 ± 0.03	0.52 ± 0.02
	Lying	0.7540	0.66 ± 0.05	0.6996	<**0.0001**	0.1350	0.65 ± 0.04	0.68 ± 0.04
	Nosing	0.2154	0.05 ± 0.01	0.2732	<**0.0001**	**0.0039**	0.03 ± 0.00	0.04 ± 0.00
	Sleeping	0.1341	0.39 ± 0.04	0.3097	<**0.0001**	**0.0077**	0.53 ± 0.03	0.47 ± 0.03
	Standing	0.1922	0.30 ± 0.05	0.6248	<**0.0001**	0.1666	0.32 ± 0.03	0.34 ± 0.03
	Suckling	0.3407	0.16 ± 0.03	0.3568	<**0.0001**	0.2430	0.18 ± 0.02	0.15 ± 0.02
	Tail wagging	0.0900	0.02 ± 0.00	<**0.0001**	<**0.0001**	**0.0006**	0.05 ± 0.00^a^	0.02 ± 0.00^b^
	Walking	0.0934	0.09 ± 0.01	**0.0347^4^**	<**0.0001**	<**0.0001**	0.08 ± 0.00	0.08 ± 0.00
	Desynchronized	0.6852	0.11 ± 0.05	**0.0404^4^**	**0.0447**	0.3329	0.14 ± 0.03	0.09 ± 0.02
	Active^7^	0.6597	0.34 ± 0.04	0.6936	<**0.0001**	0.1528	0.35 ± 0.03	0.37 ± 0.03
	Pain^8^	0.4968	0.03 ± 0.00	<**0.0001**	<**0.0001**	**0.0001**	0.06 ± 0.00^a^	0.02 ± 0.00^b^

*Values presented are the proportional means ± SE*.

a−b *Means with different superscripts in the same row differ significantly (p < 0.05)*.

1 *Only significant behavior variables are presented*.

2 *Total number of observations for each treatment group*.

3 *Significant effects are indicated in bold*.

4 *Not significant after Tukey-Kramer adjustment*.

5 *NSAID-castrated includes: 0.4 mg/kg mel cast, 1.0 mg/kg mel cast, and 6.0 mg/kg ket cast piglets*.

6 *NSAID-uncastrated includes: 0.4 mg/kg mel uncast, 1.0 mg/kg mel uncast, and 6.0 mg/kg ket uncast piglets*.

7 *Active behaviors include: nosing, suckling, walking, chewing, playing, and running*.

8 *Pain behaviors include: stiffness, trembling, spasms, tail wagging and rump scratching*.

### Piglet grimace scale

#### Comparison between NSAID-treated and control piglets

**Figure 5:**
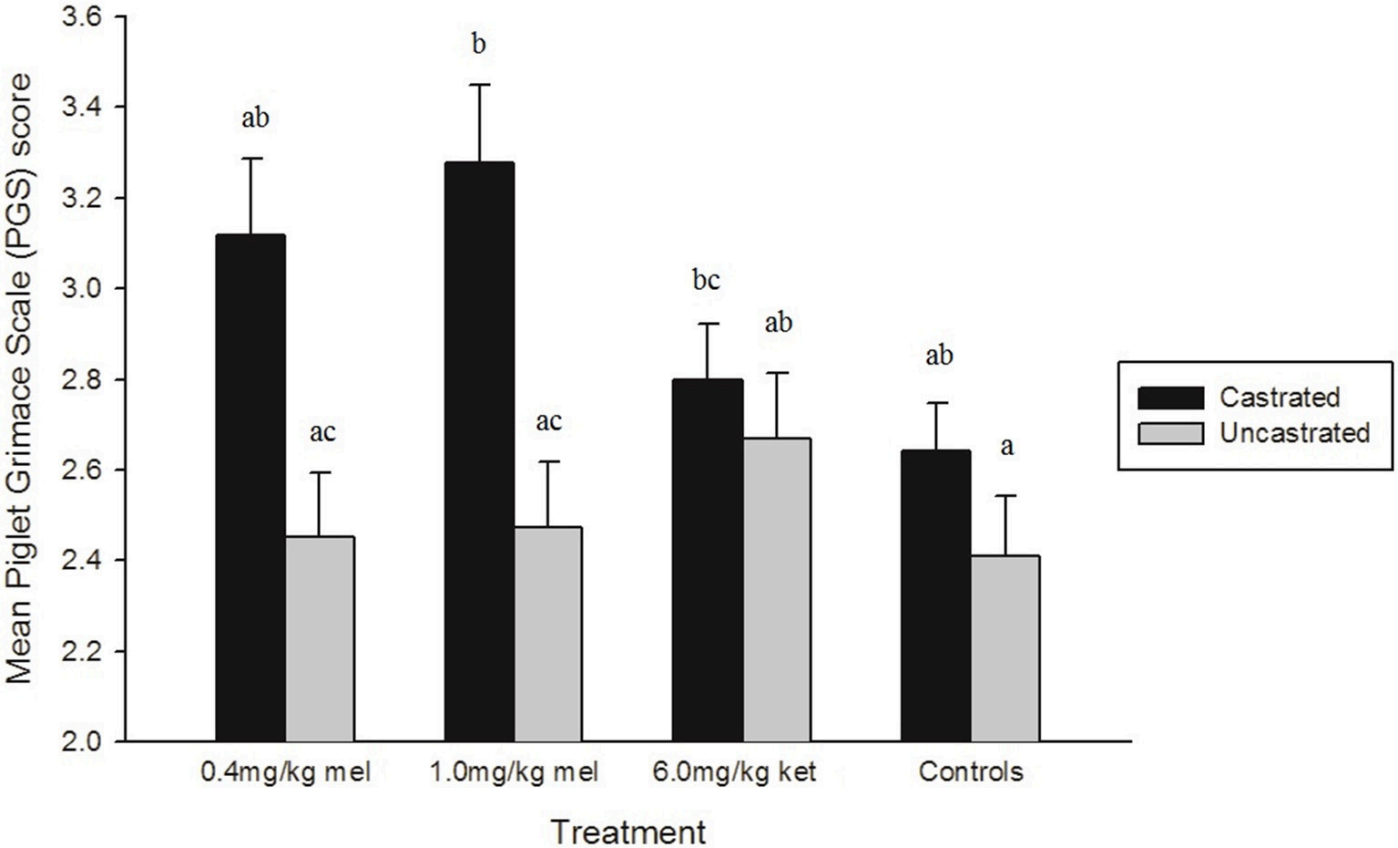
Mean Piglet Grimace Scale (PGS) scores (± SE) in each treatment group. Higher PGS scores indicate increased pain expression. Volunteers (*n* = 8) were unaware of piglet treatment, litter, and time point when scoring. Different letters (a, b, c) indicate significant differences between treatments (*p* < 0.05).

There was a significant treatment effect on PGS score (*p* = 0.0019) (Figure 5). Piglets in the sham treatment group grimaced significantly less than those in the 1.0 mg/kg MEL-castrated and 6.0 mg/kg KET-castrated piglets (*p* = 0.0101 and *p* = 0.0491, respectively), with a trend toward significance found between sham and 0.4 mg/kg MEL-castrated piglets (*p* = 0.0724). Castrated piglets treated with 1.0 mg/kg MEL grimaced significantly more than 1.0 mg/kg MEL-uncastrated and 0.4 mg/kg MEL-uncastrated (*p* = 0.0366 and *p* = 0.0256, respectively). None of the NSAID treatments significantly reduced facial grimacing in castrated piglets.

#### Comparison between castrated and uncastrated piglets

Collapsing data into castrated and uncastrated groups found, across the entire observation period, castrated piglets displayed significantly more facial grimacing than uncastrated piglets (*p* = 0.0061).

## Discussion

Meloxicam and ketoprofen are commonly used analgesics in food animals. Both NSAIDs have demonstrated efficacy in treating lameness in sows (13–15) and in reducing blood cortisol levels, heart and respiration rate in dehorned calves (16, 17). Meloxicam has previously been suggested to provide at least some analgesic effects after surgical castration in piglets (7, 18, 19); yet other studies have also found that it had no beneficial effect (4, 8). The contradictory results following NSAID use for castration have made recommendations for piglet pain control difficult (20). Two studies that found meloxicam effectively reduced behavioral indices of castration pain had a number of study design limitations that we identified. Keita et al. (7) observed piglet behavior “for a few minutes” at each time point in their study (0.5, 1, 2, 4, and 24 h post-castration) and used a very simplistic ethogram, only scoring piglets on the presence (1) or absence (0) of prostration, tremors, tail movements, and isolation. Kluivers-Poodt et al. (19) used a more detailed ethogram but employed a scan sampling method of data collection at two periods in the day (once in the morning and once in the afternoon). Both of these study methods may have resulted in a large amount of pain behaviors displayed by analgesia-treated piglets being missed and inappropriate conclusions being made with regards to drug efficacy. While both studies were sufficiently blinded to treatment, they made behavioral assessments through direct observation in the farrowing rooms, with no indication as to whether sows and piglets were habituated to the presence of an observer (7, 19). This may have impacted the behaviors observed. To address these limitations, we employed a much more comprehensive behavior scoring method (continuous observation of each piglet for 15 min per hour, at 10 time points, for a total of 2.5 h scored per piglet), and used video cameras to reduce the observer effect on animal behavior (21). We also used female researchers for all handling and technical procedures, to eliminate any potential risk of increased stress and an altered pain response in animals exposed to male researchers, as has been shown in mice (22). Our results determined that meloxicam (at either dose) and ketoprofen were ineffective in preventing or alleviating castration-associated pain in piglets. The treatment controls (piglets that were given an NSAID but were not castrated) confirmed that there are no negative behavioral side effects associated with either meloxicam or ketoprofen administration. These groups did not undergo a simulated castration because we wanted to observe the potential behavioral side effects caused by drug administration only; the stress resulting from a simulated castration may have confounded these behavioral results.

NSAIDs do not address the acute painful aspects of the castration procedure, such as the scrotal incision and tearing of the spermatic cord (23). They primarily provide analgesia by suppressing synthesis of prostaglandins responsible for inflammation post-procedure (24). Lidocaine, a local anesthetic, has been shown to decrease the pain response of piglets during castration when injected directly into the testicle (4, 18, 25, 26); however, this route of administration may be painful, and it provides minimal peri-operative analgesia (27). A multi-modal approach to pain management (meloxicam and lidocaine) is effective in reducing castration pain in piglets and calves (18, 28), but would greatly increase the castration time for each piglet and thus, may have limited practicality on-farm. A more potent drug class, such as opioids, may be required to sufficiently reduce pain; however, the feasibility of administering a controlled drug on-farm is low.

Castrated piglets demonstrated significantly more pain behaviors at 0–3 h, 6 and 7 h post-castration compared to uncastrated piglets, with castrated piglets exhibiting significantly more pain behaviors at 24 h post-castration compared to all other time points observed. This may be due to progression of the inflammatory processes, causing an increase in pain (29). Previous work found behaviors indicative of castration pain can persist in piglets beyond 24 h and some were still present 4 days after the procedure (2). 0.4 mg/kg meloxicam, administered to piglets IV, has a half-life of 2.7 h and 6.0 mg/kg ketoprofen, administered to piglets IV, has a half-life of 3.4 h (30, 31). This suggests appropriate pain management for piglets may involve more than one dose of analgesic drug following castration.

Increased activity (or restlessness) has been observed in animals experiencing pain (2, 32). This was evident in our study, with piglets at 24 h post-castration having both a significant increase in activity level and pain behaviors. The significant increase in activity 0 h post-castration may be attributed to pain from the castration procedure itself or, for piglets in the uncastrated groups, the repeated handling, IM injection or prolonged separation from the sow prior to the observation. It is possible that working in the farrowing room and castrating piglets in nearby pens may have also caused piglets already castrated (with cameras recording their behavior for scoring at 0 h) to be more alert and alter their natural behavior. Castrated piglets immediately post-procedure spent significantly more time isolated from their littermates. This behavior has previously been observed in piglets after castration as a response to pain (2, 33). An increase in tail wagging after castration has been reported in lambs, calves and piglets (2, 34–36). It has been speculated that tail wagging may signal nociceptive pain from the rear part of the body (37); however, tail wagging has also been shown to increase after dehorning calves (38). This suggests that it may serve as a less localized pain signal. It is worth noting that piglets in this study were not previously tail docked. An increase in tail wagging has been observed in piglets long after the tail docking procedure, which is thought to be attributed to tail stump hyperalgesia (39). This would not have been a cause for the significant increase in tail wagging noted here. Future work should examine tail wagging behavior as a potential indicator of pain.

Facial analysis is a novel approach to assessing pain in animals and humans. Species-specific grimace scales have been developed for mice, rats, rabbits, horses, sheep, and lambs (40–45), and involve characterizing and quantifying facial features that change in response to pain. Previous research has also described changes to piglet facial expression after a painful event (46). A cow pain face has been described (47); however, this has not been associated with a grimace scale to date. This type of pain assessment tool is non-invasive and has the potential to permit rapid detection of pain, leading to improved animal welfare if an appropriate intervention occurs promptly (48). For facial grimace scales to be validated, they must correspond well to known indicators of pain, such as behavior. In this study, we compared the PGS to pain behaviors of piglets. There was excellent agreement between the PGS and pain behaviors when assessing castrated and uncastrated pigs. When castrated and uncastrated piglets were separated into their initial treatment groups (0.4 mg/kg MEL-castrated, 0.4 mg/kg MEL-uncastrated, 1.0 mg/kg MEL-castrated, 1.0 mg/kg MEL-uncastrated, 6.0 mg/kg KET-castrated, 6.0 mg/kg KET-uncastrated, saline, and sham), the relationship between these two pain assessment tools decreased; however, every significant treatment effect found using the PGS was supported by the pain behavior results. The eight volunteers who used the PGS to score piglet faces came from various backgrounds and most had little animal experience. A more robust training session prior to having volunteers score could have been beneficial and may have resulted in stronger PGS results. Future work will focus on better training and include volunteers with greater animal experience. Overall, an increase in facial grimacing in castrated piglets corresponded to an increase in pain behaviors. This is a significant first step toward validation of the PGS as a pain assessment tool. The PGS, once validated, may be used clinically and on-farm to rapidly detect piglet pain and provide intervention. For piglets used in research, the PGS may allow for tighter endpoints (e.g., if facial grimacing moves beyond a predetermined threshold), while also providing a non-invasive measured outcome for pain assessments. In studies that assess pain interventions, reduced facial grimacing could be used as a criteria to demonstrate drug efficacy.

### Animal welfare implications and conclusion

Meloxicam at the recommended dose (0.4 mg/kg) and at a higher dose (1.0 mg/kg) and ketoprofen (6.0 mg/kg) were ineffective at alleviating surgical castration pain in piglets. NSAIDs are the most widely used drugs in Europe to reduce piglet pain and are currently being recommended by the Canadian Pork Council to comply with the recent Codes of Practice (6, 49, 50). Post-operative pain persists after castration of piglets and significantly increases at 24 h. Future work should assess a more potent drug class or drug combination to treat pain. The PGS did not detect pain as strongly as behavioral assessment, but it did correspond well with castration pain behaviors and may become a useful tool to assess piglet pain. Future validation of the PGS is needed.

## Author contributions

AV and PT conceived and designed the experiments. AV conducted the studies and analyzed the data. The manuscript was prepared and edited by AV and PT.

### Conflict of interest statement

The authors declare that the research was conducted in the absence of any commercial or financial relationships that could be construed as a potential conflict of interest.
